# Making Quality Improvements to Clinical Simulation Scenarios via Podcasting

**DOI:** 10.7759/cureus.38672

**Published:** 2023-05-07

**Authors:** Beheshta Momand, Olivia Sacuevo, Masuoda Hamidi, Flávio Vaz Machado, Adam Dubrowski

**Affiliations:** 1 Health Sciences, Ontario Tech University, Oshawa, CAN

**Keywords:** debriefing, podcasting, improving clinical scenarios, education, clinical scenarios, simulation medicine

## Abstract

Simulation is a common method for teaching and enhancing healthcare skills. Nevertheless, the construction of a simulation scenario is expensive and time-consuming and requires a great deal of effort. As a result, it is imperative that we make quality improvements to the process of scenario construction. When this is accomplished, we will be able to enhance the existing scenarios, develop new ones, and ultimately enhance these teaching tools.

Currently, publishing simulation scenarios as peer-reviewed technical reports is one way to ensure quality and global sharing of scenarios. Yet, another undiscovered potential to further improve the quality of scenarios once the peer-review process is complete is to allow the original scenario designers to reflect on their creative processes using podcasting.

This paper proposes that podcasting can be used as a supplement to the peer-review process to address this issue. Podcasting is one of the prevalent forms of media in the twenty-first century. There are currently numerous podcast channels in the healthcare simulation space. However, the majority are focused on introducing simulation experts or discussing issues in healthcare simulation, and none are focused on making quality improvements to clinical simulation scenarios with the authors. We propose to make quality improvements with scenario designers using podcasting in order to communicate information to the public and evaluate what went well and what might have been done better in order to inform future developers.

## Editorial

Over the course of the past decade, simulation has experienced a notable increase in utilization across a diverse range of fields, including military, engineering, nuclear power, aviation, and healthcare [1]. The Society for Simulation in Healthcare defines simulation as a technique that imitates situations or environments to practice, learn, evaluate, test, or understand systems and human actions [1]. Simulators (devices, programs, or systems that mimic task situations) and scenarios make up simulations (scripts or algorithms to guide interactions between students and simulators). Connecting these components requires educational theories and instructional design. Developing a simulation scenario can be time-consuming, resource-intensive, costly, and redundant. Thus, sharing "lessons learned" about the development process with the simulation community may help with reducing the human resources needed to build the scenarios.

Peer-reviewed technical reports are one way this can be done to improve scenario quality and dissemination. The peer-review process helps the original authors reflect on the creative process and identify areas for improvement for a better simulation scenario. Podcasting is another untapped way to improve scenarios after peer review. Podcasts are audio episodes that are easily accessible on computers and mobile devices. During podcasting, authors are invited to reflect on their scenario-creation process. Reflective processing may reveal insights and areas for improvement, leading to a better simulation scenario. This editorial will discuss podcasting as a method for improving healthcare simulation scenarios by conducting quality improvement (QI) with authors of previously published simulation scenarios.

The problem 

In 2015, we proposed using the Technical Reports section of the Cureus Journal of Medical Sciences, which relies on a peer-review system to enhance the quality of simulation scenarios and disseminate the information worldwide [1]. Guidelines for writing technical reports were created and published in Cureus to improve the homogeneity of simulation methods, enhance simulation quality, rate simulation scenarios, and optimize simulation efforts. Cureus allows global free publishing and downloading, promoting equity in access to simulation scenarios. Reports are assessed individually by readers with a Scholarly Impact Quotient (SIQ), embracing the collective intelligence of the Cureus community rather than the entire journal assessed based on the impact factor (IF).

Technical reports in Cureus provide useful information and a chance to evaluate the development process, but they sometimes leave out key elements like "lessons learned" before and after program execution. Allowing authors to share their experiences, dissect the simulated scenario, and discuss what worked, what did not, and what could be improved will fix this issue. This lets you learn from experts and evaluate the simulation-based education program more thoroughly. Sharing "lessons learned" about the development process with the simulation community may reduce the human resources needed to build scenarios. 

Solution 

Our editorial proposes a variety of strategies by combining quality improvement frameworks in healthcare. We specifically intend to incorporate the Institute for Healthcare Improvement (IHI), a QI tool that works in tandem with other research papers and addresses three questions: "What are we aiming to accomplish?", "How will we know whether a change is an improvement?", and "What changes can we make to make things better?" [2].

The goal of this article is to suggest using podcasting as a means of making QI to clinical simulation scenarios to improve the quality of technical reports by authors. In this editorial, we propose that we use existing simulation scenarios that were originally published in Cureus Journal of Medical Sciences, and discussing the quality of the simulations developed will help improve the quality and learn about: 1) their experience in developing the scenario, 2) some of the difficulties encountered, 3) some of the successes, 4) expressing our viewpoint, and 5) examining context-based theories to expand generalizability and update.

Example 

The paper discussed in this QI session was titled "A Post-operative Masquerade: Simulation-Based Scenario Challenging Clinical Clerks to Recognize Atypical Presentation of Myocardial Infarction" [3]. 

To listen: https://echoknowledge.simplecast.com/episodes/5-debriefing-a-simulation-scenario-on-challenging-clinical-clerks-to-recognize-myocardial-infarctions

Transcript Link: https://docs.google.com/document/d/1FflPt_H7WpQzyooXByo0QieSprxKQRRw/edit?usp=sharing&ouid=109797704740792699669&rtpof=true&sd=true

Discussion

During the above-mentioned QI scenario, Dr. Gillian Sheppard was invited to discuss the simulation scenario she and her colleagues developed in order to identify an atypical postoperative myocardial presentation. Other members including Dr. Adam Dubrowski (another author of the technical report), a Ph.D. and co-host (Beheshta Momand), and another Ph.D. student and co-host (Flavio Machado) were present to help facilitate discussion during this session. 

During the QI session, an audio clip of the scenario was first played for the listener. The scenarios were then discussed with the author in an effort to assess what transpired and what could be improved. Questions centered on an IHI model for a QI tool. The questions asked focused on the objective of the scenario, the skills being taught, the effectiveness of the simulation scenario and whether or not the skills were adopted, the ability to identify obstacles, and what changes could be made to improve the quality of the scenario if there was an opportunity to go back. To date, there are 61 simulation scenarios with 338,896 reads and a total SIQ score of 7.6. Despite the number of simulation scenarios published and reads, there has been little discussion about "lessons learned" and how to improve the scenarios. Failure to learn from past simulation scenarios can lead to repeating errors, costly mistakes, and no improvements. QI of clinical simulation scenarios can be very beneficial.

There are currently channels in the field of simulation and podcasting. The Center for Medical Simulation, Simulation in Healthcare Education, and Simulcast are just some of the podcasts that deal with healthcare simulation, but none of them are devoted to actually QI pre-existing clinical scenarios. Consequently, we think that our podcast channel Echo Knowledge can introduce this new idea of QI which could help us reevaluate and improve the existing scenario designs.

In addition, in this paper, we suggested using an IHI model of the QI tool which includes Plan, Do, Study, Act (PDSA) phases for continuous improvement. However, more emphasis is given to planning and execution, and there may be other frameworks that could assess the quality of simulation scenarios that are more focused on program evaluation or reflexivity. The Program Evaluation Framework involves stages such as goal identification, model development, evaluation design, and data-based decision-making [4]. Reflexivity promotes self-awareness and critical reflection, helping to identify biases and facilitate open communication during QI [5].

Conclusion 

In conclusion, improving the quality of clinical simulation scenarios is vital if one wishes to see increased levels of success. This is because simulations that are constructed without any sort of guidance or framework can be time-consuming, costly, and inconsistent. Currently, peer-reviewed technical reports are shared on Cureus to improve scenario quality and dissemination; however, it does not address the lessons learned. In an effort to disseminate knowledge and enhance the quality of the scenario, we discussed in this paper how podcasting (a popular media platform) could be used to facilitate QI. During this QI, we will discuss what went well, what could have been improved, and what lessons were learned during the development of the shared scenario.

A major challenge encountered during this process was the difficulty in scheduling interviews with busy physicians, long after the report had been published. To overcome this issue in future podcast sessions, we plan to contact authors promptly after publication to schedule interviews in advance. We believe that these efforts will generate valuable insights and enhance the quality of our reports. Through these efforts, we hope to gain valuable insights and improve the quality of these reports. 

“The more you know about the past the better prepared you are for the future” - Theodore Roosevelt. 
